# Compressed Sensing-Based MRI Reconstruction Using Complex Double-Density Dual-Tree DWT

**DOI:** 10.1155/2013/907501

**Published:** 2013-06-06

**Authors:** Zangen Zhu, Khan Wahid, Paul Babyn, Ran Yang

**Affiliations:** ^1^Department of Electrical and Computer Engineering, University of Saskatchewan, Saskatoon, SK, Canada S7N 5A9; ^2^Department of Medical Imaging, University of Saskatchewan and Saskatoon Health Region, Saskatoon, SK, Canada S7N 0W8; ^3^School of Information Science and Technology, Sun Yat-Sen University, Guangzhou, Guangdong 510006, China

## Abstract

Undersampling *k*-space data is an efficient way to speed up the magnetic resonance imaging (MRI) process. As a newly developed mathematical framework of signal sampling and recovery, compressed sensing (CS) allows signal acquisition using fewer samples than what is specified by Nyquist-Shannon sampling theorem whenever the signal is sparse. As a result, CS has great potential in reducing data acquisition time in MRI. In traditional compressed sensing MRI methods, an image is reconstructed by enforcing its sparse representation with respect to a basis, usually wavelet transform or total variation. In this paper, we propose an improved compressed sensing-based reconstruction method using the complex double-density dual-tree discrete wavelet transform. Our experiments demonstrate that this method can reduce aliasing artifacts and achieve higher peak signal-to-noise ratio (PSNR) and structural similarity (SSIM) index.

## 1. Introduction

Magnetic resonance imaging (MRI) is one of the major imaging modalities in use today. Compared to computed tomography (CT), MRI has advantages in imaging soft tissues. However, its relatively long imaging time remains a great challenge for clinical application, often limiting its application. Significant efforts have focused on faster data collection as well as reducing the amount of data required without degrading image quality. For example, parallel imaging [1–3] exploits redundancy in *k*-space by introducing multiple receiver channels, mitigating the aliasing artifacts caused by a reduced sampling rate. Recently, compressed sensing based MRI (CS-MRI) allows high quality reconstruction from undersampled data by enforcing the pseudo-sparsity of images in a predefined basis or dictionary, such as the traditional two-dimensional (2D) separable wavelet transform or total variation [4]. However, these basis sets may not provide sufficient sparse representation. The discrete wavelet transform (DWT), for example, has three major disadvantages: shift sensitivity [5], poor directionality [6], and lack of phase information [7, 8]. For these reasons, traditional DWTs fail to capture regularities of contours, since they are not able to sparsely represent one-dimensional singularities of 2D signals [9]. Therefore, improvements can be obtained by mitigating some of these disadvantages simultaneously.

In this paper, we propose an improved compressed sensing method for MR imaging by utilizing the double-density dual-tree DWT [10]. The use of complex wavelet transforms for compressed sensing was first proposed in [11]. The authors in [11] used dual-tree complex wavelet transform (DT-CWT) as a sparsifying transform, which only has wavelets oriented in six directions. But as natural images exhibit smooth regions that are punctuated with edges at several orientations, dual-tree complex wavelet transform may fail to sparsely represent the geometric regularity along the singularities, which require higher directional selectivity. Other contour-based transforms, such as contourlets [12], have also been investigated. But they can only sparsely represent the smooth contours but not the points in images [13, 14]. In this paper, we propose one possible solution by using a newly developed multiresolution tool, double-density dual-tree transform, which may provide sufficient sparse representation for MR images with different features. Total variation is also exploited as a penalty in the reconstruction formulation to suppress noise. Note that in [11], the authors applied their method to radial trajectories. In this paper, its variant on Cartesian sampling is used for comparison, namely, CS DT-CWT. To differentiate between the original compressed sensing based MRI algorithm [4] and the improved version, we will denote the original algorithm in [4] as CS and our proposed method as iCS (improved compressed sensing MRI).

The rest of this paper is organized as follows. In Section 2, we briefly review principles of MRI and then discuss the design of our proposed algorithm. In Section 3, we will present the experimental results of our algorithm in comparison with some other algorithms. Finally, a brief conclusion will be drawn.

## 2. Theory and Method

### 2.1. Magnetic Resonance Imaging (MRI)

 MRI signal is generated by the proton in hydrogen atoms, the main component of the human body. Each proton in an atomic nucleus possesses a fundamental spin. Since protons are charged particles, when a human body is placed in an strong static magnetic field *B*_0_, protons will align themselves with the magnetic field, yielding a net magnetic moment precessing around *B*_0_. This net magnetic precession is termed *Larmor precession*. The frequency of Larmor precession is proportional to the applied magnetic field strength as defined by

(1) $$\begin{array}{c} f=\gamma B_{0}, \end{array}$$

where *γ* is a constant (42.57 MHz/T) [15]. Next, a radiofrequency (RF) pulse is applied perpendicular to *B*_0_. If the frequency of the applied pulse is equal to the Larmor frequency, the net magnetic moment will tilt away. Once the RF signal is removed, the protons realign themselves such that the net magnetic moment is again aligned around *B*_0_. The protons return to equilibrium by emitting RF signal, which is then captured by a conductive field. This measurement is reconstructed to obtain gray-scale MR images.

To produce a 3D image, a gradient magnetic field, *G*_*z*_, is added to *B*_0_ so that the Larmor frequency changes linearly in the axial direction, *z*. Hence, an axial slice can be selected by choosing a specific Larmor frequency of that slice. Additionally, two gradient magnetic fields, *G*_*x*_ and *G*_*y*_, are applied causing the resonant frequencies of the protons to vary according to their positions in the *x*-*y* plane. As a result, the signal is encoded in three dimensions. If the signal is fully sampled at the Nyquist rate, a 2D inverse Fourier transform is then used to transform the encoded image to the spatial domain. Consider the following:

(2) $$\begin{array}{c} x=F^{-1}y, \end{array}$$

where *y* is the measurements from scanner, which is also called *k*-space data, *F* is the Fourier transform matrix, and *x* is the desired MR image. But in the real world, downsampling may be needed for some applications, such as to fit the scans into one-breath hold or to enable real time-imaging.

### 2.2. Features of Complex Double-Density Dual-Tree Wavelet

**Figure 1:**
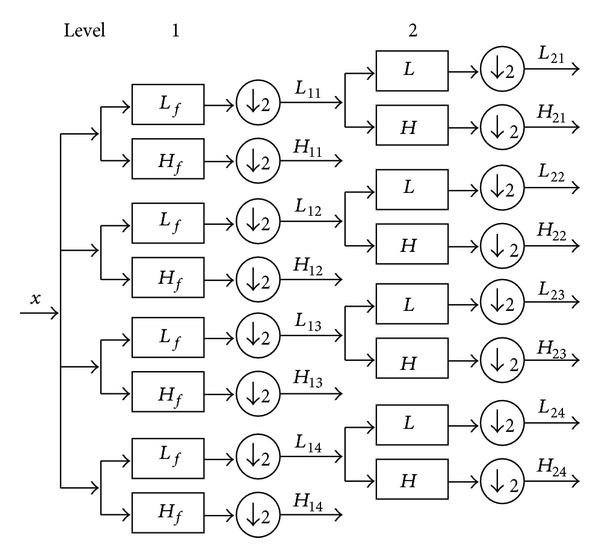
Two levels of 2D complex double-density dual-tree wavelet transform.

The 1D double-density discrete wavelet transform (DD-DWT) is based on one scaling function (i.e., low pass) and two different wavelets (i.e., high pass) where one wavelet is a half-sample shift of the other. The 2D transform applies the 1D transform alternately to the rows and column, giving nine subbands, where one is a low pass subband and the remaining eight subbands become eight wavelet filters. Thus, the 2D complex double-density dual-tree discrete wavelet transform (CDDDT-DWT) is implemented by using four 2D DD-DWT in parallel with different filter banks for rows and columns separately [10].  Figure 1 shows the process of two levels of the transform. *L*_*f*_ and *H*_*f*_ are the filter banks of the first level decomposition, which represent one scaling filter and eight wavelet filters. *L* and *H* are the filter banks for the second and remaining levels of decomposition. Therefore, four low pass subbands (*L*_11_–*L*_14_) and 32 high pass subbands (*H*_11_–*H*_14_) are produced after one level of the transform. As a result, 32 wavelets are created by the sum and difference on each pair of subbands.

**Figure 2:**
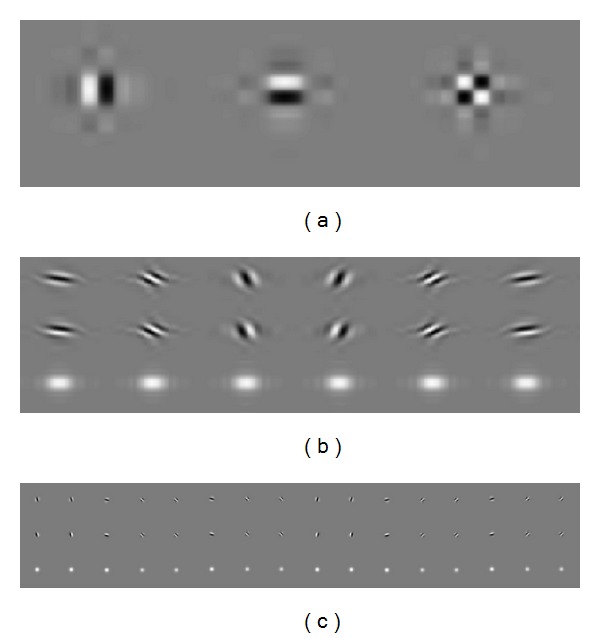
Impulse responses of (a) 2D DWT, (b) 2D DT-CWT, and (c) 2D CDDDT-DWT, as illustrated at level of 4 of the transforms.

Figure 2 shows the impulse responses of these transforms. The figures are obtained by setting all the wavelet coefficients to zero, for the exception of one wavelet coefficient in each of the high pass subbands of one level. We then take the inverse wavelet transform [16–18]. Therefore, if the transform has more directional wavelets, then fewer coefficients are needed to represent a given geometric object.  Figure 2(a) shows the typical wavelets associated with the 2D wavelet transform. Obviously, 2D wavelet transform can resolve only three spatial-domain feature orientations: vertical, horizontal, and diagonal. In addition, the third wavelet does not have a dominant orientation, which is the main cause of artifact (checker board pattern). Therefore, traditional 2D separable wavelet fails to sparsely represent geometric structures, such as edges [19].

In Figure 2(b), six orientations (±15°, ±45°, ±75°) are obtained by 2D DT-CWT. By contrast, complex double-density dual-tree wavelet transform has 32 wavelets oriented in 16 different angles (see Figure 2(c)). The 16 wavelets shown in the first (second) row can be interpreted as the real (imaginary) parts of the set of 16 complex wavelets and the third row are the magnitudes of the 16 complex wavelets. As we can see, the orientations are richer and finer. Additionally, all of the wavelets are free of checker board pattern. As a result, we can get more accurate representation for the geometric regions. 

**Figure 3:**
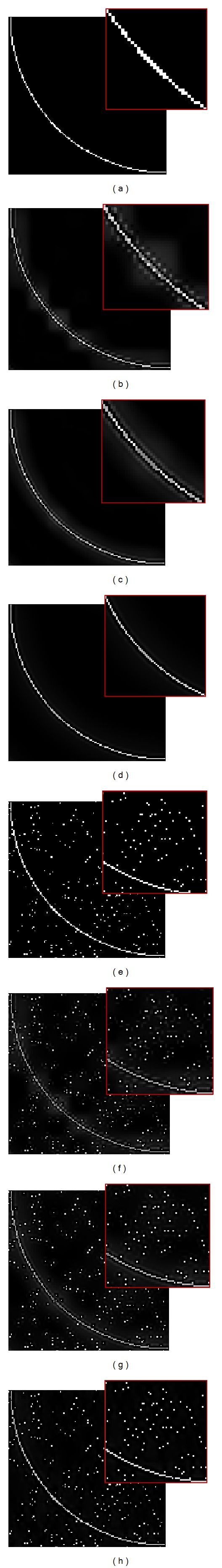
Improved directionality of complex double-density dual-tree wavelet transform: (a) original test image; reconstructed image using only the lowest level coefficients of (b) DWT; (c) DT-CWT; (d) CDDDT-DWT; (e)–(h) results of the same experiment with noisy image (zoomed-in at the right corner). Gray level is normalized between [0, 1] for all images and 4-level transform is used for all experiments.

In Figure 3, we illustrate this benefit with two example images. The first image group only contains a curve purposely designed to demonstrate the improved directionality property of CDDDT-DWT. The second image group contains impulse (or salt and pepper) noise. We reconstruct the test images using the first stage of transform coefficients. As we can see from Figures 3(b) and 3(f), there are blocking artifacts in DWT reconstructions since it can only accurately represent vertical and horizontal lines. Because DT-CWT has more directional wavelets, the reconstructed curve looks smoother and artifacts are reduced. But six orientations (Figure 2(b)) are not sufficient to accurately represent this curve as it contains all directions. The reconstructions from CDDDT-DWT are much closer to the original ones. This is because it has more wavelets which are strongly oriented at 16 different angles. One may also conclude that 16 orientations are sufficient to represent any geometric object with high precision. Compared to the curve, the points are better reconstructed by all the three transforms. As stated in the introduction section, wavelet transforms are good at capturing point singularities [20]. From this example, we can see that CDDDT-DWT can sparsely represent both contours and points, indicating its superiority for these tasks.

### 2.3. Reconstruction Algorithm Using Compressed Sensing

The problem of undersampling *k*-space data actually leads to an underdetermined system of linear equations. One way to improve performance is to incorporate a prior knowledge into the reconstruction process which is based on the idea of sparsity in compressed sensing (CS) [21–23]. The essence of CS is that a signal, which in our case is the image *x*, can be completely reconstructed with a high probability with far less samples than required by conventional Nyquist-Shannon sampling theorem, if the image has a sparse/compressible representation in a transform domain Φ, such that most entries of the vector Φ*x* are zero or close to zero.

The entire process consists of three steps [24]: encoding, sensing, and decoding. In the first step, the object image *x* of size *n* is encoded into a smaller vector *y* = *F*_*u*_ *x* of a size *m* (*m* < *n*) by the system matrix. *F*_*u*_ denotes the Fourier matrix associated with some undersampled trajectory while *y* is the corresponding undersample *k*-space data. Directly solving the underdetermined linear system will yield numerous solutions. As we assume the image is approximately sparse in CDDDT-DWT, that is, *α* = Φ*x*, has few elements with relatively large magnitudes, a solution is possible. Then the second step is obtaining the undersampled *k*-space data *y* from the imaging system. Incorporating the sparsity prior knowledge into the process of image reconstruction, the third step is to recover *α* (and thus *x*) by solving the following constrained optimization problem:

(3) $$\begin{array}{c} \arg\underset{x}{\min}{||\Phi x||}_{1}\;\text{subject}\;\;\text{to}\;\;{||F_{u}x-y||}_{2}<\varepsilon , \end{array}$$

where *ε* is a parameter controlling the data consistency. It has been mathematically proven that if the image has only *k* entries with relatively large magnitudes, the order of *k*log⁡(*n*) measurements is sufficient to accurately reconstruct *x* via the *ℓ*_1  _ norm minimization procedure with high probability. As we have noticed from Figure 3, noise may be also reconstructed by complex double-density dual-tree wavelet. Therefore, in our proposed algorithm, we include the total variation (TV) as a penalty because it was shown that it is efficient in suppressing the noise in the reconstructed image [24]. The constrained problem in (3) can also be converted into an unconstrained problem, giving rise to our proposed iCS model:

(4) $$\begin{array}{c} \arg\;\underset{x}{\min}\lambda_{1}{||\Phi x||}_{1}+\lambda_{2}{||x||}_{\text{TV}}+{||F_{u}x-y||}_{2}^{2}, \end{array}$$

where two regularization factors *λ*_1_ and *λ*_2_ are introduced to leverage the cost function's emphasis on the transform *ℓ*_1  _ penalty, TV penalty, and the data fidelity term. The selection of regularization factor has been an interesting area of research in the field of regularized iterative methods [25, 26]. A large *λ*_2_ tends to suppress image gradient and make the reconstructed image look smooth, losing point-like features. In our study, we chose the optimized regularization parameters *λ*_1_ and *λ*_2_ for all methods for a fair comparison. The discussion will be given in next section. The TV term of an image in this work is defined as follows:

(5) $$\begin{array}{c} {||x||}_{\text{TV}}=\int \left|\nabla x\right|dx. \end{array}$$

In a discrete version, (5) becomes

(6) $$\begin{array}{c} {||x||}_{\text{TV}}=\sum_{i,j}\sqrt{{\left(x_{i+1,j}-x_{i,j}\right)}^{2}+{\left(x_{i,j+1}-x_{i,j}\right)}^{2}}. \end{array}$$

To speed up the implementation, we exploit a fast implementation of CDDDT-DWT [27]. Since (4) poses an unconstrained convex optimization problem, we propose solving it using a nonlinear conjugate gradient descent algorithm that is similar to [4]. It has been shown in [28] that the iterative algorithm in our study has better performance than the algorithm in [4]. *J*(*x*) is the cost function as defined in (4). The iterative algorithm starts with a zero-filling Fourier reconstruction.

The conjugate gradient requires the computation of ∇*J*(*x*) which is

(7) $$\begin{array}{c} \nabla J\left(x\right)=\lambda_{1}\nabla {||\Phi x||}_{1}+\lambda_{2}\nabla {||x||}_{\text{TV}}+2F_{u}^{*}\left(F_{u}x-y\right). \end{array}$$

As the *ℓ*_1_ norm and total variation term (5) is the sum of absolute values, the absolute values, however, are not smooth functions, and as a result (7) is not well defined. In [4], Lustig et al. approximated the absolute value with a smooth function, $\left|x\right|\approx \sqrt{x^{*}x+\xi }$, where *ξ* is a positive smoothing parameter. Then the gradient becomes $d\left|x\right|\approx x/\sqrt{x^{*}x+\xi }$.

We adopt this idea in our implementation. In particular, a smoothing factor *ξ* = 10^−15^ is used. The algorithm of the proposed iCS method is shown in Algorithm 1. 

## 3. Numerical and Experimental Results

**Figure 4:**
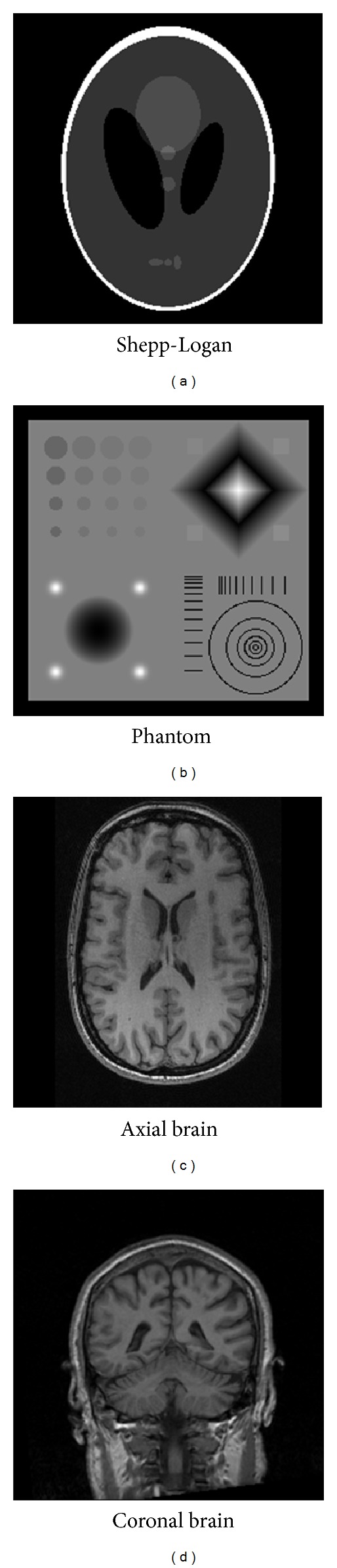
Datasets in this study.

In this section, we report our experiments to evaluate and validate the proposed algorithm. There are five sets of experiments. In the first two experiments, numerical phantoms and simulated *k*-space data were used to study the performance of our algorithm. The third and fourth experiments used real data collected from real scanners. In the fifth experiment, we manually add noise to the *k*-space data of the fourth data set. The first phantom that we consider is the discrete Shepp-Logan, which contains geometrical structure and directional-oriented curves. The second phantom is purposely designed to be nonsparse under total variation domain and contains features difficult to reproduce with partial Fourier sampling [29]. In such way, we can clearly see how the CDDDT-DWT affects the image quality. The third dataset was performed on a 1.5T GE Signa Excite scanner. This T2-weighted dataset of the brain was acquired axially using a FSE sequence [30]. The final dataset was a coronal section of a brain obtained from a T1-weighted brain scan [31]. To have a clear differentiation between these datasets, we name these four datasets as Shepp-Logan, phantom, axial brain, and coronal brain, respectively, as shown in Figure 4. All images are of size 256 × 256.

**Figure 5:**
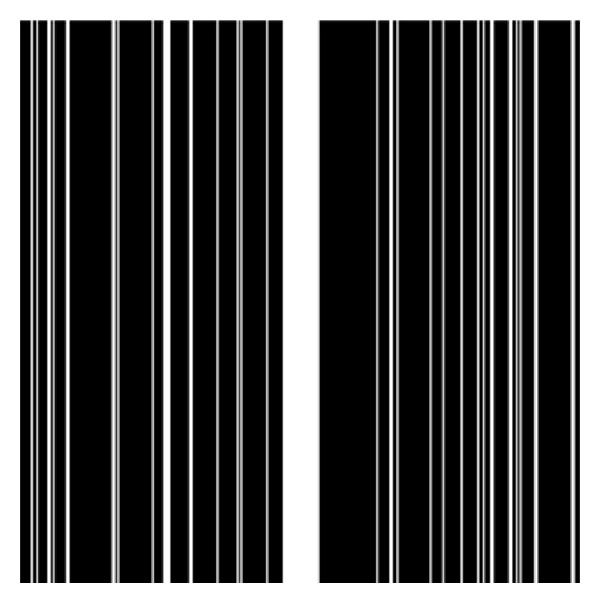
Sampling pattern at 0.2 sampling rate. Undersampling is done along the phase direction.

Undersampling *k*-space is simulated with random phase encoding in Cartesian sampling. The sampling density decreases according to a power of distance from the origin. It should be pointed out that the proposed method also works with non-Cartesian sampling pattern, such as spiral and radial trajectories, although these are not shown. Reconstructions were performed at different sampling rates: 0.1, 0.15, 0.2, 0.25, 0.3, 0.35, and 0.4. A sampling pattern with sampling rate 0.2 is shown in Figure 5 where white bar means that the data is sampled and black means otherwise (i.e., not sampled).

The reconstructed data was quantitatively evaluated in terms of peak signal-to-noise rate (PSNR) and structural similarity (SSIM) index [32]. PSNR measures the difference between the reconstructed image $\hat{x}$ and original image *x*, which is defined by

(8) $$\begin{array}{c} \text{PSNR}=20\;\text{lo}{\text{g}}_{10}\left(\frac{\text{MAX}}{\sqrt{\text{MSE}}}\right), \end{array}$$

where $\text{MSE}=(1/n)\sum_{i=0}^{n}{\left(x_{i}-{\hat{x}}_{i}\right)}^{2}$ and MAX is the maximum possible pixel value of the image.

The structural similarity (SSIM) index is highly effective for measuring the structural similarity between two images. Suppose *ρ* and *t* are local image patches taken from the same location of two images that are being compared. The local SSIM index measures three similarities of the image patches: the similarity of luminances *l*(*ρ*, *t*), the similarity of contrasts *c*(*ρ*, *t*), and similarity of structures *s*(*ρ*, *t*). Local SSIM is defined as

(9) S(ρ,t)=l(ρ,t)·c(ρ,t)·s(ρ,t)    =(2μρμt+C1μp2+μt2+C1)(2σρσt+C2σp2+σt2+C2)(2σρt+C3σρσt+C3),

where *μ*_*ρ*_ and *μ*_*t*_ are local means, *σ*_*ρ*_ and *σ*_*t*_ are local standard deviations, and *σ*_*ρt*_ is cross-correlation after removing their means. *C*_1_, *C*_2_, and *C*_3_ are stabilizers. The higher the value of SSIM, the higher image quality is delivered.

**Figure 6:**
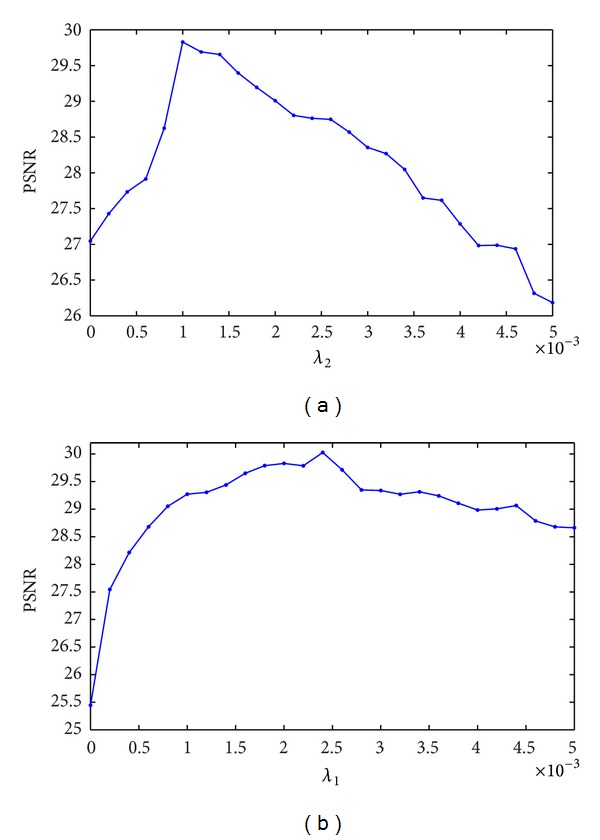
Analysis to find the optimum regularization parameters (for Coronal Brain data at sampling rate 0.25). (a) *λ*_2_ when *λ*_1_ = 0.002 for iCS method; (b) *λ*_1_ when *λ*_2_ = 0.001 for iCS method.

In this paper, three methods will be compared under identical conditions. These three methods have three different sparsity transforms Φ in (4). The first method uses the discrete wavelet transform (shown as CS) [4]; the second method uses dual-tree complex wavelet transform (shown as CS DT-CWT) [33]; the proposed method uses complex double-density dual-tree wavelet transform (CDDDT-DWT, denoted by iCS for the rest of the paper). A 4-level of Daubechies-4 wavelet transform was used for CS method. Reconstruction was done under the same conditions such as iterative algorithm and sampling pattern as the accuracy depends on the selection of optimum regularization parameters. We have used the last dataset, Coronal Brain, as an example to show the methodology of determining the optimal parameters. For each algorithm, we alternately plotted the PSNR against one parameter keeping the other fixed. We started by setting *λ*_1_ = 0.002.  Figure 6(a) shows that the highest PSNR is obtained when *λ*_2_ is 0.001. Then we set *λ*_2_ = 0.001 and searched the optimal value for *λ*_1_ that gives the highest PSNR, as shown in Figure 6(b). Thus, we used this recurring process to determine the optimum values of *λ*_1_ and *λ*_2_. Similar search was conducted for the other two compared methods and all datasets. The optimal values of these parameters are shown in Table 1. 

**Figure 7:**
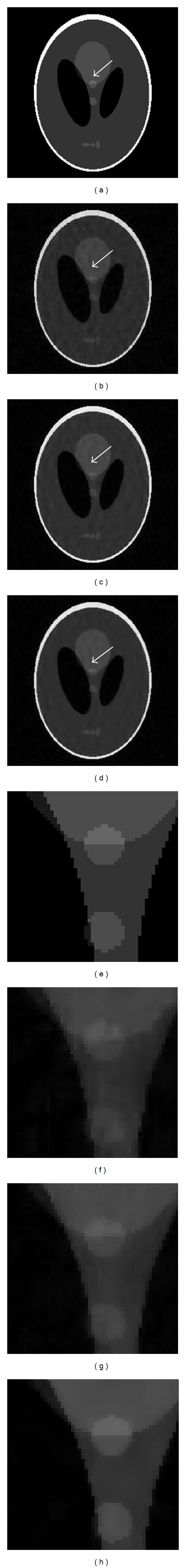
Original Shepp-Logan image and experimental results. (a) Original Shepp-Logan image. Reconstruction at sampling rate 0.2 by (b) CS; (c) CS DT-CWT; (d) iCS technique; (e), (f), (g), and (h) are detail views of region outlined by arrow in (a), (b), (c), and (d), respectively.

We begin with the phantom experiments.  Figure 7(a) is an image reconstructed from the fully sampled data. Figures 7(b), 7(c), and 7(d) are reconstructions by CS method, CS DT-CWT, and our proposed method iCS, respectively. The weakness of the traditional separable wavelet transform in representing two-dimensional singularities, for example, curves and edges, is visualized with the reconstructed Shepp-Logan images. As we have seen that separable wavelet transform can resolve only three spatial-domain feature orientations. Therefore, the ellipses are not sparsely represented, causing substantial artifacts in the reconstructed images (Figure 7(b)). The artifacts become more serious when the size of ellipse is reduced. For example, two small ellipses around center (as indicated by white arrows) are faded away from the CS reconstruction. To see it clearly, detailed zoom-ins are shown in Figures 7(e)–7(h). Moreover, the CS reconstruction shows substantial artifacts in smooth region. By contrast, the proposed method reconstructs the image with higher visual image quality. From Figures 7(e) to 7(h), we can see that, with more directional wavelets, the ellipses are better reconstructed. The reconstruction by complex double-density dual-tree wavelet transform has the best visual image quality. Additionally, the two small ellipses could be clearly recognized; see Figure 7(h). 

**Figure 8:**
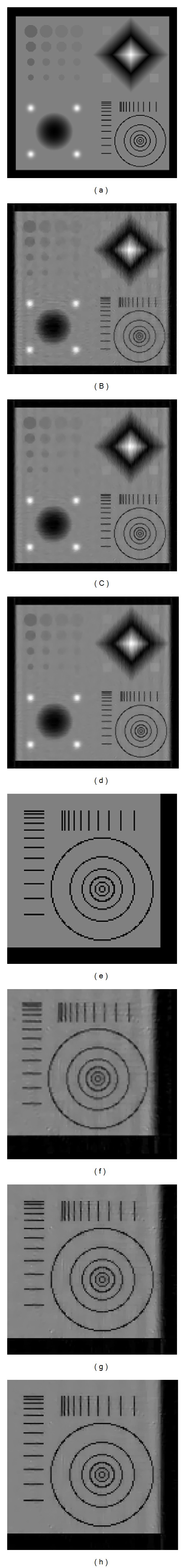
Original phantom image and experimental results. (a) Original phantom image. Reconstruction at sampling rate 0.2 by (b) CS; (c) CS DT-CWT; (d) iCS technique; (e), (f), (g), and (h) are detail views of region outlined by arrow in (a), (b), (c), and (d), respectively. Gray level is normalized in [0, 1].

Similar results can be obtained from the second phantom experiment. From Figure 8(b), we can see significant aliasing artifacts in the CS reconstruction. These artifacts are caused by the use of nonideal low pass and high pass filters in the traditional separable wavelet transform. Additionally, from the detailed zoom-in (Figure 8(f)), we can see that CS cannot reconstruct those closely placed lines separately. By contrast, CS DT-CWT and iCS can separate these lines. One possible reason for this phenomenon may be that the wavelets of DW-CWT and CDDDT-DWT are finer than those of DWT see Figure 1. Therefore, small size objects are better reconstructed with finer wavelet filters. We can see reduced artifacts from Figures 8(f) to 8(g). This is evident that our method leads to a better reconstruction with higher spatial resolution.

**Figure 9:**
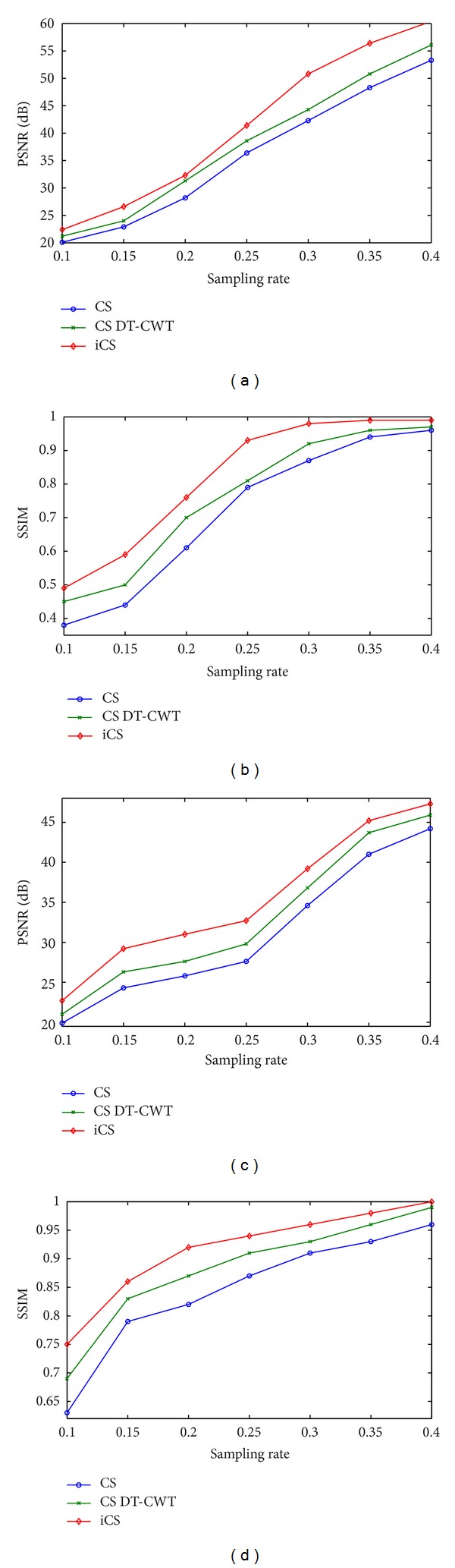
Plots of PSNR and SSIM versus different sampling rates. (a) PSNR of Shepp-Logan. (b) SSIM of Shepp-Logan. (c) PSNR of phantom dataset. (d) SSIM of phantom dataset.

In order to see how the results vary with the sampling rates, experiments were also performed at sampling rates of 0.1, 0.15, 0.25, 0.3, 0.35, and 0.4. The plots of PSNR and SSIM versus different sampling rates for the phantom simulations are shown in Figure 9. At sampling rate 0.25, the PSNRs and SSIMs increase dramatically. The PSNR of the Shepp-Logan image by our proposed method is 41.5 dB, and structural similarity is well above 0.93. There is no visual difference between the reference and reconstructed images using the proposed method. On the other hand, reducing the sampling rate below 0.2, reconstructed images are obviously blurred and their quality is not acceptable. One possible reason for the failure of compressed sensing methods is that the initial image is too bad at such low sampling rates.

**Figure 10:**
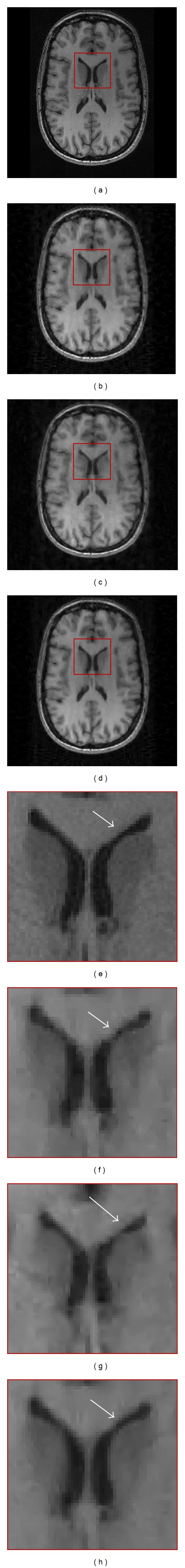
Original axial brain image and experimental results. (a) Original axial brain image. Reconstruction at sampling rate 0.2 by (b) CS; (c) CS DT-CWT; (d) iCS technique; (e), (f), (g), and (h) are detail views of region outlined by arrow in (a), (b), (c), and (d), respectively.

**Figure 11:**
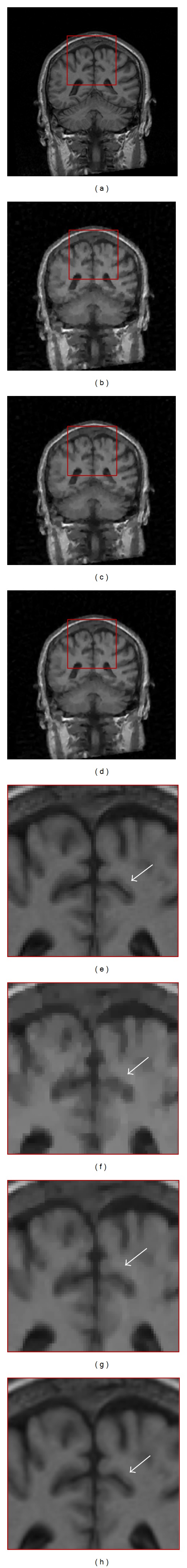
Original coronal brain image and experimental results. (a) Original coronal brain image. Reconstruction at sampling rate 0.25 by (b) CS; (c) CS DT-CWT; (d) iCS technique; (e), (f), (g), and (h) are detail views of region outlined by arrow in (a), (b), (c), and (d), respectively.

Next we consider the in vivo experiments without noise added purposely. Figures 10 and 11 show the reconstructed images where (a), (b), (c), and (d) are original images, CS reconstructions, CS DT-CWT reconstructions, and our iCS reconstructions, respectively. Note that although both CS reconstructions are able to reduce the undersampling artifacts significantly, the images obtained by CS contain significant blocking artifacts along directional edges. To emphasize this point, detail views of the reconstructed images are also displayed in these figures. This is because the diagonal wavelet of traditional wavelet transform does not have a dominant orientation see Figure 1(a). As a result, it can not represent the curves efficiently, leading to considerable artifacts along edges. Dual-tree complex wavelet transform can resolve six orientations, both of which have a dominant orientation, and thus the image quality is greatly improved by CS DT-CWT. In contrast, since complex double-density dual-tree wavelet has improved directional selectivity and finer wavelets than DWT and DT-CWT, the proposed method preserves more edges and small structures. Therefore, the images obtained using our proposed method are much closer to the original images.  Figure 10(h) and Figure 11(h) indicate that edges are sharper in the reconstructed image using our proposed method.

**Figure 12:**
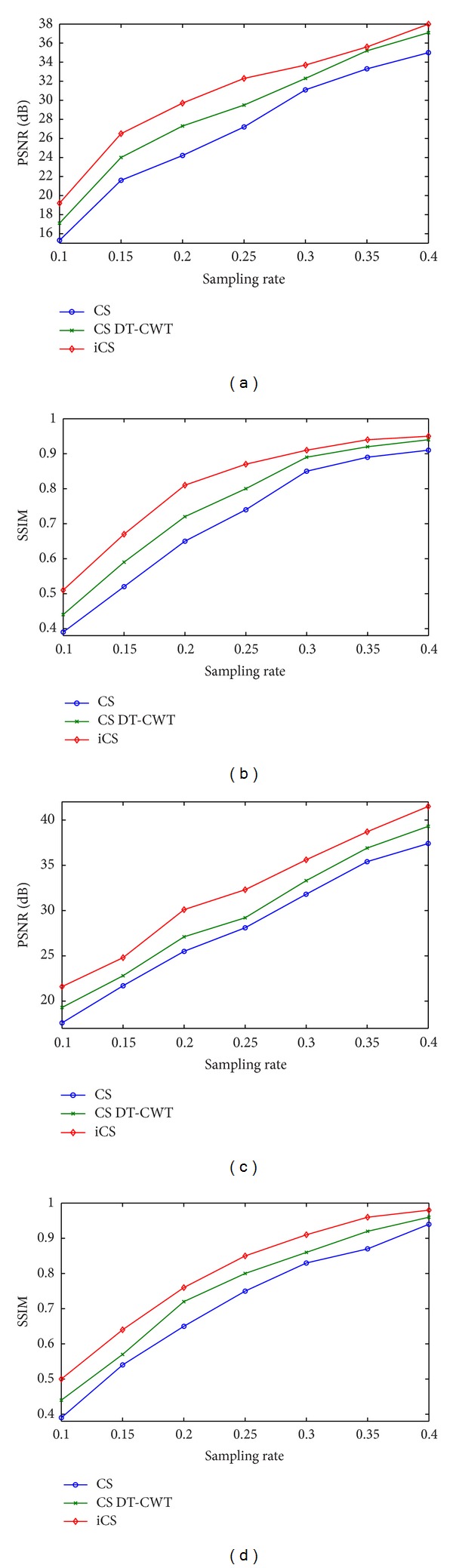
Plots of PSNR and SSIM for different sampling rates: (a)-(b) axial brain; (c)-(d) coronal brain.

The evaluation matrices (PSNRs and SSIMs) of the reconstructed images are also plotted against the sampling rates in Figure 12. Consistent increase of reconstruction accuracy using the proposed method is observed. Figure 12 also indicates that PSNRs and SSIMs of reconstructions by our proposed method in all cases are higher than those of other methods. One should also note that the original CS method can produce an accurate reconstruction given a sufficient sampling rate. For instance, the results in [4] showed that CS can produce high quality brain image without artifacts at sampling rate 0.42 (2.4 acceleration). This conclusion is confirmed by our in vivo experiments. Note that the PSNR of CS method is well above 35 dB and SSIM is as high as 0.9 at sampling rate 0.4. But at low sampling rates, reconstructed image is not satisfactory. As a result, traditional wavelet transform fails to provide sparse representation, causing artifacts. Our proposed complex double-density dual-tree wavelet is able to capture those geometric features that are not captured by other transforms, producing a higher quality of image. The results of this test confirm that our proposed method outperforms the original CS method in preserving edges and suppressing undersampled artifacts. 

**Figure 13:**
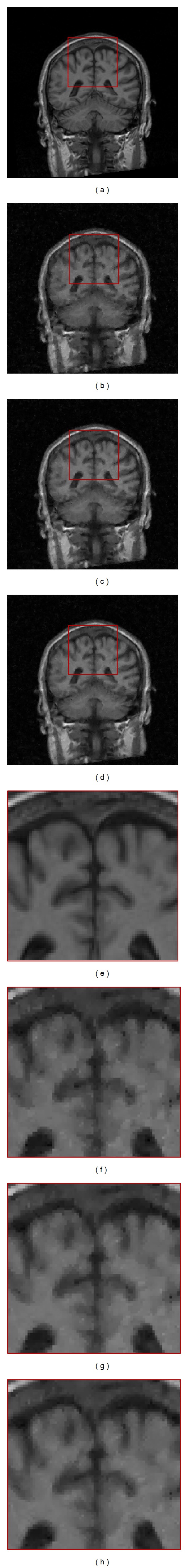
Original coronal brain image and experimental results with noisy data. (a) Original coronal brain image. Reconstruction at sampling rate 0.25 by (b) CS; (c) CS DT-CWT; (d) iCS technique; (e), (f), (g), and (h) are detail views of region outlined by arrow in (a), (b), (c), and (d), respectively.

Finally, we reconstructed the coronal brain image using imperfect *k*-space data to test the robustness of the proposed method. Additive Gaussian white noise *e* of relative magnitude ||*e*||_2_/||*Fx*_ture_||_2_ = 0.05 was purposely added to the *k*-space data. The results are shown in Figure 13. The optimum parameter selections are also shown in Table 1. From the table, we can see that the parameter *λ*_2_ is bigger when noisy data is used for reconstruction for all the methods. This is because TV regularization is good at suppressing noise. Larger *λ*_2_ can better suppress noise. But if *λ*_2_ is too large, the reconstructed image will be oversmooth, losing low contrast or detailed information. The PSNR and SSIM are summarized in Table 2. Although the image quality is reduced, the proposed method can still maintain the best results.

From the various results presented in this section, proposed method delivers higher PSNR and SSIM than other methods. However, the computation time of CS-based methods including the proposed algorithm is about 70 seconds. Development of faster algorithms for solving problem (4) will be pursued in the future work.

## 4. Conclusion

In this paper, an improved compressed sensing MRI method is proposed. The directionality property of complex double-density dual-tree wavelet transform is investigated. Considering the edge features and quality, we employ objective measurements to evaluate the performance of our approach. Simulation results on phantom and in vivo data demonstrate that the proposed method can better reconstruct the edges and reduce undersampled artifacts than traditional CS-MRI method does. In our implementation, we use the nonlinear conjugate gradient iterative method to solve the unconstrained optimization problem. Further effort is needed to utilize a more advanced iterative method to improve reconstructed precision and reduce computational time for real time-imaging. Extension of this work with the nonconvex optimization is being considered to further improve the reconstruction. 

## Figures and Tables

**Algorithm 1:**
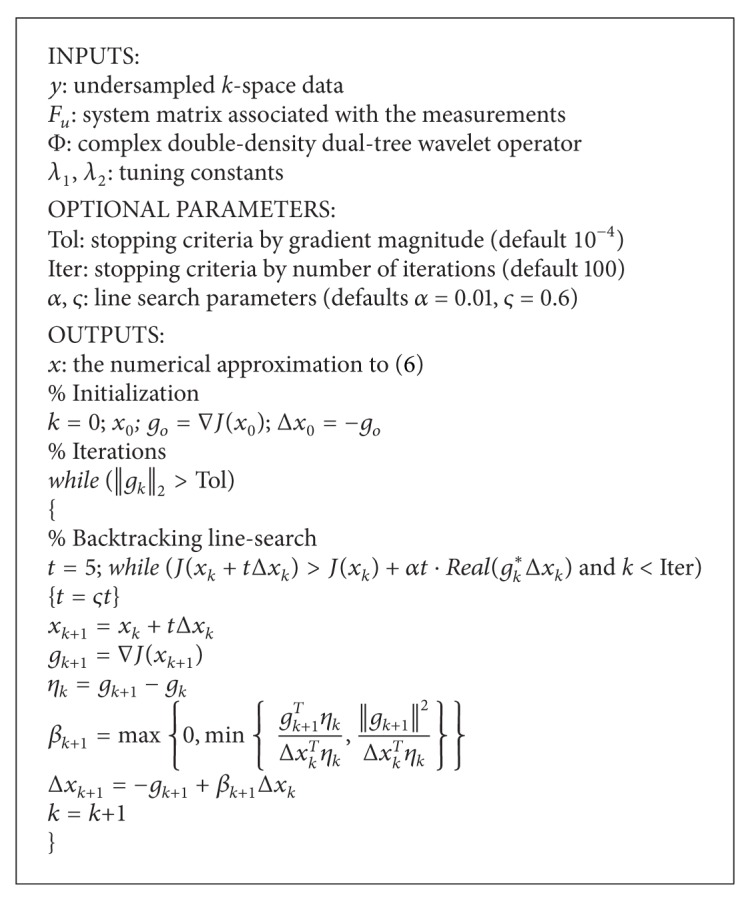


**Table 1:** Optimal parameter selections at 0.25 sampling rate for coronal brain data and 0.2 for other data.

Dataset	CS [4]		CS DT-CWT		iCS	
	*λ*_1_	*λ*_2_	*λ*_1_	*λ*_2_	*λ*_1_	*λ*_2_
Shepp-Logan	0.0014	0.0024	0.001	0.0026	0.0012	0.0024
Phantom	0.001	0.0016	0.001	0.0018	0.0016	0.0016
Axial brain	0.0014	0.001	0.0012	0.0012	0.0016	0.0008
Coronal brain	0.0014	0.0006	0.0014	0.0012	0.0024	0.001
Coronal brain with noise	0.0014	0.001	0.0012	0.0018	0.0018	0.0012

**Table 2:** PSNR and SSIM of coronal brain image with noisy data at 0.25 sampling rate.

Image	Reconstruction methods	PSNR (dB)	SSIM
Coronal brain	CS [4]	24.9	0.61
	CS DT-CWT	27.1	0.69
	iCS	29.0	0.75
